# Development of a multivariable prediction model for early revision of total knee arthroplasty – The effect of including patient-reported outcome measures

**DOI:** 10.1016/j.jor.2021.03.001

**Published:** 2021-03-11

**Authors:** J.D. Andersen, S. Hangaard, A.A.Ø. Buus, M. Laursen, O.K. Hejlesen, A. El-Galaly

**Affiliations:** a Department of Health Science and Technology, Faculty of Medicine, Aalborg University, Denmark; b Steno Diabetes Center North Denmark, Aalborg University Hospital, Aalborg, Denmark; c Orthopaedic Research Unit, Aalborg University Hospital, Aalborg, Denmark; d Department of Clinical Medicine, Aalborg University, Aalborg, Denmark

**Keywords:** TKA, Machine learning, Knee osteoarthritis, Prediction model, Revision

## Abstract

**Background:**

Revision TKA is a serious adverse event with substantial consequences for the patient. As revision is becoming increasingly common in patients under 65 years, the need for improved preoperative patient selection is imminently needed. Therefore, this study aimed to identify the most important factors of early revision and to develop a prediction model of early revision including assessment of the effect of incorporating data on patient-reported outcome measures (PROMs).

**Material and methods:**

A cohort of 538 patients undergoing primary TKA was included. Multiple logistic regression using forward selection of variables was applied to identify the best predictors of early revision and to develop a prediction model. The model was internally validated with stratified 5-fold cross-validation. This procedure was repeated without including data on PROMs to develop a model for comparison. The models were evaluated on their discriminative capacity using area under the receiver operating characteristic curve (AUC).

**Results:**

The most important factors of early revision were age (OR 0.63 [0.42, 0.95]; *P* = 0.03), preoperative EQ-5D (OR 0.07 [0.01, 0.51]; *P* = 0.01), and number of comorbidities (OR 1.01 [0.97, 1.25]; *P* = 0.15). The AUCs of the models with and without PROMs were 0.65 and 0.61, respectively. The difference between the AUCs was not statistically significant (*P =* 0.32).

**Conclusions:**

Although more work is needed in order to reach a clinically meaningful quality of the predictions, our results show that the inclusion of PROMs seems to improve the quality of the prediction model.

## 1 Introduction

Knee osteoarthritis (OA) is one of the most common forms of OA and is estimated to affect 260 million people worldwide.^1^ It is estimated that hip and knee OA alone are accounting for 20 million years lived with disability worldwide and thus have considerable impact on the individual patient.^1^, ^2^, ^3^ Knee OA often results in severe pain, disability and deterioration in health-related quality of life,^4^^,^^5^ translating into substantial healthcare costs and productivity losses for individuals and society.^6^^,^^7^ There are different treatment options for knee OA aiming to restore function and relief pain with total knee arthroplasty (TKA) considered the most effective treatment for end-staged multicompartmental knee OA.^8^, ^9^, ^10^ TKA is one of the most frequently performed orthopedic surgeries in Denmark with nearly 7.000 primary TKAs performed in 2018.^11^ In comparison, more than 700.000 TKAs are performed in USA annually and approximately 100.000 TKAs are performed in the UK.^12^ Worldwide rates of both primary and revision TKA are rising, primarily due to the increased longevity of the population and the obesity epidemic.^4^^,^^12^^,^^13^ Internationally, consistent revision rates have been reported but the number of revisions is expected to rise as an inevitable consequence of the rising number of primary TKAs.^9^^,^^14^ In USA, prediction models based on registry data are projecting the demand for primary and revision TKA to increase by 600% by 2030 reaching more than 3.5 million TKAs and more than 270.000 revisions.^15^^,^^16^ Revision TKAs are more complicated, more expensive and associated with both reduced implant-survival and inferior patient-reported outcomes (PRO) when compared to primary TKA.^17^^,^^18^ An increasing number of patients under 65 years are estimated to have a TKA in the future. These patients will be more likely to have revision and maybe even re-revision due to their life expectancy.^19^, ^20^, ^21^ A previous study has shown that the risk of revision TKA is 2.5 times greater in those under 65 years than in those above 65 years at the time of surgery.^8^ More importantly, patients under 55 years at the time of surgery have a 5-fold increased risk of revision three years postoperatively compared with those above 75 years.^8^^,^^21^ Machine learning (ML) and prediction modeling is increasingly used within the field of orthopedic surgery and earlier attempts to predict potential outcomes of TKA have been made in terms of patient-satisfaction, range of movement and length of stay.^22^, ^23^, ^24^, ^25^ Additionally, a recent study by El-Galaly et al. developed different prediction models for early revision TKA using different ML-algorithms based on national registry data. However, this study did not include any patient-reported outcome measures (PROMs) or information on comorbidity, which has recently been suggested to potentially improve the performance of these models.^26^^,^^27^ Therefore, it is yet to be investigated whether it is possible to develop a clinical meaningful prediction model for early revision in patients undergoing TKA while including data on PROMs and comorbidity. Moreover, it is yet to be explored whether including PROMs data yield a better performing model than one without PROMs. Furthermore, because of primary TKA being increasingly considered for patients under 65 years, there is a strong need to identify predictor variables and define risk groups leading to improved preoperative patient selection in clinical practice. Therefore, this study aimed to 1) identify the most important factors of early revision in patients undergoing TKA and 2) develop a prediction model for early revision in patients undergoing TKA including assessment of the effect of incorporating data on PROMs and comorbidity in the model.

## 2 Material and methods

The study is reported in accordance with the Transparent Reporting of a multivariable prediction model for Individual Prognosis Or Diagnosis (TRIPOD) guidelines^28^ and the Strengthening the Reporting of Observational studies in Epidemiology (STROBE) statement.^29^ The study was approved by the Danish Data Protection Agency before data collection (entry no. 2008-58-0028).

### 2.1 Study design and cohort

The study was a descriptive correlational and predictive study based on a cohort of patients undergoing primary TKA between November 11, 2014 and December 20, 2016 at the Department of Orthopedic Surgery at Aalborg University Hospital, Denmark. Eligible patients were required to 1) have underwent primary TKA for primary or secondary knee OA 2) be aged ≥30 at the time of surgery, and 3) have completed the PROM preoperatively. In cases where a patient had bilateral surgery during this period, only data from the first surgery was included in the analysis. All patients were followed for two years with substitution, removal or addition of an implant or part of an implant considered as revision.

### 2.2 Data source

We used combined registry data obtained from the Danish Knee Arthroplasty Registry (DKR) and from the Department of Orthopedic Surgery at Aalborg University Hospital's administrative outcome database “Jointbase”. The Jointbase was developed in 2013 to monitor the quality of treatment and care of patients and prospectively collects PRO-data on patients undergoing TKA.^30^ The DKR is a national registry that prospectively collects information on all knee arthroplasties inserted in Denmark, and the completeness of the database has been above 96% for primary arthroplasties and 93% for revision arthroplasties since 2011.^11^^,^^31^

### 2.3 Predictor variables

All predictor variables were selected from patient demographics and characteristics, PROMs and clinical data on surgery-related factors based on previous studies and clinical reasoning.

Patient demographics and characteristics included in the analysis were age (grouped into decades), gender, body mass index (BMI), pain at rest and activity measured by visual analogue scale (VAS), diabetes mellitus, and number of comorbidities. PROMs included in the analysis were preoperative Oxford Knee Score (OKS)^32^ and preoperative EuroQol-5D (EQ-5D).^33^ OKS is a validated joint-specific instrument consisting of 12 patient-administered questions evaluating pain and function in relation to the knee. The final score is summed up on a scale ranging from 0 to 48 (worst-best). An overall score was calculated between 0 and 48 and subsequently converted to an overall score between 0 and 100, which was used as a continuous variable.^32^^,^^34^ EQ-5D is a validated instrument for measuring generic health-related quality of life. The instrument consists of two components: 1) the EQ VAS by which patients assess their perceived health status ranging from 0 (worst imaginable health status) to 100 (best imaginable health status), and 2) the EQ-5D descriptive system which evaluates perceived health status within five dimensions: Mobility, self-care, usual activities, pain/discomfort, and anxiety/depression.^33^^,^^34^ The EQ-5D provides an overall index score (EQ-5D index) between 0 and 1 (worst-best) for a patient's health status, which was used as a continuous variable. Clinical data on surgery-related factors included in the analysis were previous surgery in the same knee, duration of surgery, and length of stay.

### 2.4 Missing data

In total <1% of total values in the dataset were missing with the highest percentage of missing values in previous surgery in the same knee (1.3%). Complete-case analysis was performed and cases with missing data were excluded from the dataset prior to further data analysis.

### 2.5 Study outcome

The outcome for the prediction model was early revision TKA. This was characterized as revision for any indication within two years of the primary TKA. In accordance with the DKR revision was defined as removal, exchange, or addition of an implant or part of an implant.^11^

### 2.6 Prediction model

Multiple logistic regression (LR) with forward selection of variables was used as a prediction model. To internally validate the model, stratified 5-fold cross-validation was performed. The procedure was as follows: Firstly, simple LR analysis was performed to identify potentially important variables. All potential predictor variables with a *P* < 0.25 were included in further analysis. Secondly, multiple LR with forward selection of variables was applied to the dataset to identify the most important features of the model. Thirdly, the dataset was shuffled randomly and then split into five equally sized samples with each sample containing the same amount of outcome observations. Fourthly, the model was trained with four of the samples working as training set and the remaining sample working as test set. This procedure was repeated five times until each sample of the five folds had been used as test set once and to train the model four times. Finally, the overall evaluation score was retained and used as an estimate of the performance of the model. Additionally, the exact same procedure was performed but without the data on PROMs (i.e., EQ-5D and OKS) to build a model for comparison. The performance of the model was evaluated on discrimination and calibration using receiver operating characteristic (ROC) curve, area under the curve (AUC)^35^, ^36^, ^37^, ^38^ and the Hosmer-Lemeshow goodness-of-fit score. Difference between the AUCs was compared as described by Hanley and McNeil.^39^ The discriminative capacity of prediction models are ranked by their AUCs as follows: Excellent (0.9–1.0), good (0.8–0.89), fair (0.7–0.79), poor (0.6–0.69), and failed (0.5–0.59).^40^ An AUC ≥0.70 was chosen as cut-off to define a clinical meaningful model.

### 2.7 Statistical analysis

Continuous data are presented with mean and standard deviation (SD) and categorical data with frequencies (n) and percentages (%). Comparison of continuous variables were conducted with unpaired *t*-tests and categorical variables with chi-square or Fisher's exact test. Multiple LR analyses were performed using forward selection of variables and the stepwise criteria of the probability-of-F to enter and remove variables were set at 0.05 and 0.10, respectively. The regression beta coefficients (B), odds ratios (OR), 95% confidence intervals (CI), and p-values (*P*) were calculated. LR diagnostics were performed to assure basic test assumptions were met. To assure absence of multicollinearity, analysis of potential collinear variables was made from the observed variance inflation factor. A p-value of <0.05 was considered statistically significant. All statistical analyses were computed using SPSS Statistics (version 27; IBM Corp., Armonk, New York) and MATLAB (version R2020a, MathWorks, Massachusetts, USA).

## 3 Results

### 3.1 Sample size

**Fig. 1:**
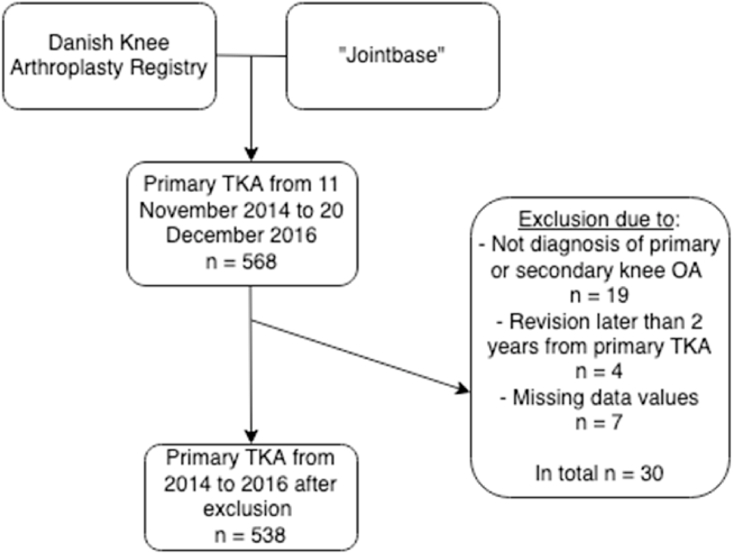
Flow-chart illustrating the retrieval and exclusion process of the dataset.

A total of 568 patients were identified through the DKR and Jointbase to have underwent primary TKA between November 11, 2014 and December 20, 2016. Due to other diagnosis than primary or secondary knee OA 19 patients were excluded. Due to revision later than two years from primary TKA, four patients were excluded, and seven patients were excluded due to missing data values. Among the 568 patients, a total of 538 patients met the eligibility criteria and were included in the cohort for analysis. Full overview of the exclusion process is presented in Fig. 1.

### 3.2 Sample characteristics

A full list of characteristics is presented in Table 1. Patients were aged between 37 and 91 (mean age 67.5) and 55% were female. The majority of the patients had 1-2 comorbidities (51%). The percentage of revision TKA was 4.5% with infection and aseptic loosening as the major indications for revision.

**Table 1:** Sample characteristics.

Characteristic	Data
Total number of patients, *n* (%)	538 (100%)
Age in years, mean (SD)	67.5 (9.6)
Gender, *n* (%)	
Male	244 (45%)
Female	294 (55%)
BMI, mean (SD)	29.9 (5.5)
Duration of surgery, mean (SD)	64 min (19.4)
Length of stay, mean (SD)	1.9 (1.3)
Revision within 2 years, *n* (%)	24 (4.5%)
Revision cause, *n* (%)	
Infection	9 (38%)
Aseptic loosening	6 (25%)
Instability	3 (13%)
Pain	1 (4%)
Unknown	5 (20%)
Previous surgery in same knee, *n* (%)	
Yes	143 (27%)
No	395 (73%)
Pain preoperative, mean (SD)	
VAS rest	48.4 (25.3)
VAS activity	68.4 (21.4)
Diabetes mellitus, *n* (%)	
Yes	174 (32%)
No	364 (68%)
Number of comorbidities, *n* (%)	
None	203 (38%)
1–2	273 (51%)
3+	62 (11%)
**Total**	538 (100%)
OKS 0–100 preoperative, mean (SD)	43.4 (14.7)
EQ-5D index preoperative, mean (SD)	0.62 (0.18)

Table 1 - Overview of characteristics. Categorical variables are presented as frequencies (n) and percentages (%). Continuous variables are presented as mean and standard deviation (SD). BMI = Body mass index, VAS = Visual analogue scale, OKS = Oxford knee score.

### 3.3 Predictors of early revision

Potential predictor variables and simple LR analyses of predictor variables associated with early revision are presented in Table 2. After excluding predictor variables with *P* > 0.25, seven variables were included in the multiple LR analysis. Using forward selection of variables, the best predictors of early revision were age (OR 0.63 [0.42, 0.95]; *P* = 0.03), preoperative EQ-5D index (OR 0.07 [0.01, 0.51]; *P* = 0.01) and number of comorbidities (OR 1.01 [0.97, 1.25]; *P* = 0.15). These results are presented in Table 3.

**Table 2:** Simple Logistic Regression Analysis for identifying predictors of early revision.

Predictors	B	OR	*P*	OR 95% CI
Gender^a^	0.19	1.21	0.64	[0.53, 2.75]
BMI	0.01	1.01	0.89	[0.93, 1.09]
Age	−0.52	0.59	0.01*	[0.39, 0.88]
Pain rest (VAS)	0.01	1.01	0.35	[0.99, 1.03]
Pain activity (VAS)	0.01	1.01	0.52	[0.99, 1.03]
Diabetes mellitus^b^	−0.15	0.85	0.73	[0.34, 2.10]
Previous surgery in same knee^c^	0.53	1.70	0.22*	[0.73, 3.98]
OKS score 0-100	−0.04	0.96	0.02*	[0.93, 0.99]
EQ-5D index	−2.53	0.08	0.01*	[0.01, 0.55]
Length of stay	0.22	1.25	0.05*	[1.00, 1.57]
Duration of surgery	0.02	1.02	0.01*	[1.00, 1.03]
Number of comorbidities	0.08	1.09	0.16*	[0.97, 1.22]

Table 2 – Simple Logistic Regression analysis for identifying predictors of early revision. B = regression beta coefficients; OR = odds ratio; CI = confidence intervals. P = p-value. *P < 0.25.

a Male = 1; female = 0.

b Yes = 1; no = 0.

c No = 1; yes = 0.

**Table 3:** The Best Predictors of early revision.

Predictors	B	OR	*P*	OR 95% CI
Age	−0.46	0.63	0.03	[0.42, 0.95]
EQ-5D index	−2.70	0.07	0.01	[0.01, 0.51]
Number of comorbidities	0.01	1.01	0.15	[0.97, 1.25]

Table 3 – The best predictors of early revision. B = regression beta coefficients; OR = odds ratio; CI = confidence intervals; P = p-value.

### 3.4 Prediction model

**Fig. 2:**
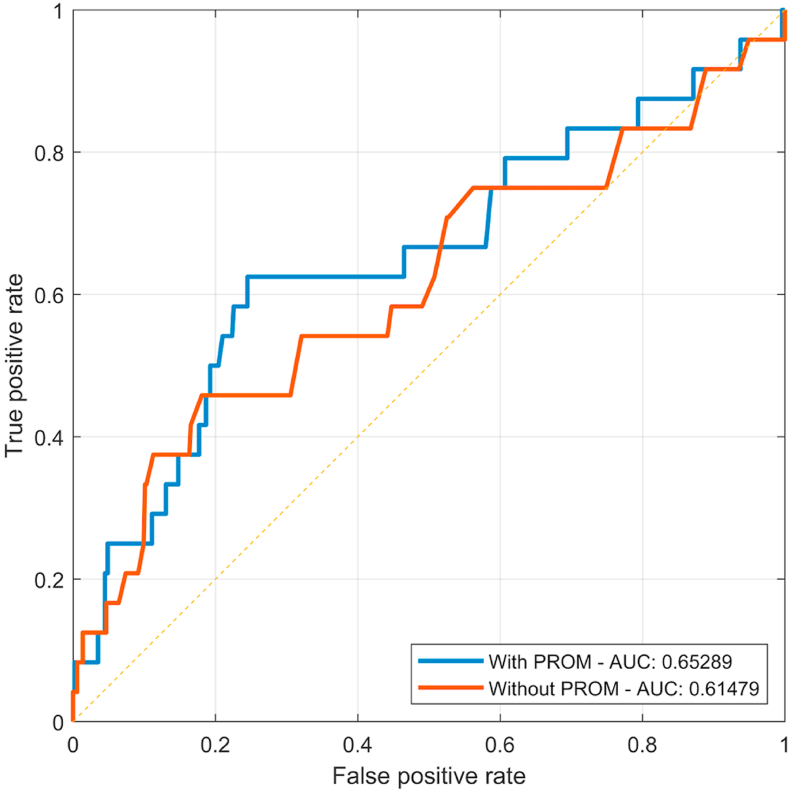
Receiver Operating Characteristic (ROC)-curve. Area Under the Curve (AUC) with and without PROMs. With PROMs AUC = 0.65. Without PROMs AUC = 0.61. Blue line = with PROMs, red line = without PROMs, yellow line = reference line (no model).

ROC-curve and AUC of the models with and without PROMs are presented in Fig. 2. As depicted in the ROC-curve, the model including PROMs had an AUC = 0.65, while the model without PROMs had an AUC = 0.61. The difference between the AUCs was not statistically significant (*P* = 0.32). The Hosmer-Lemeshow test score for goodness-of-fit was *X*^*2*^ = 4.41, *P =* 0.82 for the model with PROMs and *X*^*2*^ = 4.38, *P =* 0.82 for the model without PROMs.

## 4 Discussion

This study aimed to identify the most important predictors of early revision, and to develop a prediction model capable of predicting early revision in patients undergoing TKA including assessment of the effect of incorporating data on PROMs. To the authors’ knowledge, this is the first attempt to develop such a model including both data on PROMs and comorbidity. This study demonstrated that the best predictors of early revision were age, preoperative EQ-5D, and number of comorbidities. As depicted in the ROC-curve, neither of the prediction models did reach the predefined threshold for a clinical meaningful model (AUC = 0.65 and 0.61).

The findings in the present study are predominantly complementing findings from other studies reporting that the risk of revision is increasing with younger age, primarily due to a combination of implant wear and increased level of physical activity compared to older counterparts.^19^^,^^41^^,^^42^ A low preoperative EQ-5D score has not previously been linked with increased risk of revision but has been reported to be associated with a reduced chance of returning to desired physical activity following TKA.^43^ Although physical inactivity might not cause wearing of the implant it could instead lead to suboptimal rehabilitation of the knee, which could further lead to a poor outcome following TKA ultimately necessitating revision. The finding that an increasing number of comorbidities was a predictor of revision is generally in accordance with previous studies, which reports on poor outcomes following TKA because of the increased risk of postoperative complications.^44^, ^45^, ^46^ For example, diabetes is a known risk factor for postoperative infections, and infection was the most common cause of early revision in the present study.^47^ However, diabetes alone was not a predictor of revision in the present study. Surprisingly, predictors such as BMI and previous surgery in the same knee were also not associated with early revision in the present study. These findings are equivocal with some studies reporting that BMI is not a predictor of worse outcome following TKA,^48^, ^49^, ^50^ while others are suggestive of BMI being associated with increased risk of revision.^51^^,^^52^ The finding that previous surgery in the same knee was not associated with a worse outcome following TKA is contrary to what other studies report.^53^^,^^54^

In the present study, the prediction models’ discriminative capacity were categorized as “poor” with an AUC of 0.65 and 0.61^40^ and thereby only showed marginally better ability to distinguish between those with and without the outcome than no model. This performance is complementing previous studies attempting to predict orthopedic-related factors, also applying LR models.^55^, ^56^, ^57^ A recent study by El-Galaly et al. failed to build a clinical meaningful prediction model for early revision despite the use of different ML-algorithms such as random forest, gradient boosting model and supervised neural network.^26^ However, future studies might still benefit from combining PROMs data and these more advanced types of models, even though no sophisticated ML-algorithm can fully overcome the fact that revisions are rare.^27^ No threshold was established in the present study to classify revision and no revision. Therefore, an accurate report of sensitivity and specificity was not possible. It should be noted, however, that for an arbitrarily fixed true-positive rate of 60%, for example, it would result in a false-positive rate at 25% in the model with PROMs and a false-positive rate of 50% in the model without PROMs, thus demonstrating the effect of adding PROMs to the model.

One strength of the present study was its reliance on representative and contemporary data obtained from the DKR, a national clinical quality database and part of the Danish Clinical Quality Program, and from a single center specialized hospital.^31^ Prediction models relying on routinely collected data are more readily implementable in clinical practice.^58^ Another strength was the careful selection of predictor variables that was based on both previous studies and clinical reasoning, and the combination of national registry data and PROMs. Potential limitations include the limited sample size and lack of dealing with the imbalanced dataset (514 unrevised TKAs vs. 24 revised TKAs). A large class imbalance might increase the risk of the model treating all the underrepresented classes as background noise or simply assume that all revised patients were unrevised and thereby remain a high accuracy.^59^ One way to deal with this is by using under- or oversampling techniques to inflate the imbalance combined with increasing the sample size which will make it less prone to imbalance problems.^59^ Many clinical predictors were used in the present study, yet it did not contain information on intraoperative components, surgical technique, or surgeon volume that potentially could be important explanatory variables in terms of revision surgery.^54^ Inclusion of annual volume of arthroplasties on surgeon-level instead of hospital-level might have improved the performance of the model in the present study. However, this information was not accessible through the DKR. Despite this, future studies might benefit from combining patient-related factors with intraoperative or postoperative factors as these possibly have a stronger correlation to revision surgery. Although intraoperative or postoperative information is not of great value during the preoperative patient selection, it might improve the planning and monitoring of the patient's postoperative course. Another limitation is the lack of external validation of the model due to no access to such data. Without external validation it is not possible to estimate the performance of the model on new data, and therefore not possible to evaluate the generalizability and transferability of the model.^60^ To compensate for this, internal validation with stratified 5-fold cross-validation was performed, which have been shown to minimize the risk of bias in datasets with a limited sample size.^38^ Finally, the study may be limited by the use of stepwise selection of variables at significance level. This approach may cause selection bias and overestimate regression coefficients as a result of overfitting. Overfitting leads to less accurate predictions, especially in small datasets or with weakly predictive variables.^38^^,^^61^ However, despite the use of stepwise selection, a relatively high number of variables, and the limited sample size, the present results are not estimated to be a direct consequence of overfitting due to the poor AUC. Instead, it is more likely to be a result of weakly predictive variables with poor correlation to the outcome.

Prediction models as a health technology could have important future applications in clinical practice by assisting clinicians in their pre and postoperative planning and shared decision making.^24^^,^^62^^,^^63^ The combination of ML and data is essential in terms of predicting the risk of certain outcomes and have already provided predictions regarding opioid use after spinal surgery,^64^ different PROMs following TKA,^24^^,^^55^ patient-satisfaction and length of stay following TKA.^22^^,^^25^ This combination yields a unique opportunity to improve preoperative patient selection, monitoring, and identification of risk groups. The field of knee arthroplasty have a long tradition of data collecting through national registries, which provide readily accessible data from large population-based cohorts.^65^ Thus, making it well suited for application of ML to develop prediction models readily useable within Denmark and easily transferable to countries with similar registries.

## 5 Conclusions

The present study developed and evaluated a prediction model aimed to predict early revision TKA and can be seen as a first step towards determining the most important factors of early revision. Although more work is needed in order to reach a clinically meaningful quality of the predictions, our results show that the inclusion of PROMs seems to improve the quality of the model.

## Funding information

This research did not receive any specific grant from funding agencies in the public, commercial, or not-for-profit sectors.

## Declarations of competing interest

None.
